# Face-Ache

**Published:** 1844-03

**Authors:** 


*Face-Ache.*—This common affection, so often supposed to be excited by a diseased tooth, although the latter fails to be detected—a rheumatic, chronic kind of pain, wholly different from that of tic-douloureux,—is often speedily curable by *muriate of ammonia.* This salt should be given in doses of half a drachm, dissolved in water, three or four times daily. About four doses will be sufficient to test the potency of the remedy. At other times the iodide of potassium, in five or six-grain doses, is quickly effective towards a cure. The efficiency of the latter remedy renders it probable that the affection is of the nature of periodical inflammation.

*Dr. Watson's Lectures.*

